# Expression of Prostanoid EP3 Receptors in Oral Squamous Epithelium and Oral Squamous Cell Carcinoma

**DOI:** 10.1155/2015/602929

**Published:** 2015-02-09

**Authors:** Muhammad Kashif, Muhammad Ishfaq, A. H. Nagi

**Affiliations:** ^1^Department of Morbid Anatomy and Histopathology, University of Health Sciences, Lahore 54000, Pakistan; ^2^Oral & Maxillofacial Surgery Department, Nishtar Institute of Dentistry, Multan 60000, Pakistan

## Abstract

*Objectives*. To carry out a descriptive analysis of the expression of the EP3 receptors of PGE2 in different histological grades of OSCC and adjacent normal epithelium. *Material and Methods*. A total of 46 patients presenting with various histological subtypes and grades of OSCC were recruited from Maxillofacial Surgery Department of Nishtar Institute of Dentistry Multan. Microscopically tumour subtyping and histological grading according to Anneroth's grading system were carried out. Immunohistochemical staining with rabbit polyclonal EP3 receptor antibody was performed and sections were scored for intensity and proportion of positive adjacent squamous epithelial and tumour cells. *Results*. Out of 46 patients *n* = 28 (60.9%) were well differentiated, *n* = 15 (32.6%) were moderately differentiated, and only *n* = 3 (6.5%) were poorly differentiated. All *n* = 46 cases of OSCC were positive for EP3 receptor antibody, *n* = 14 (30.4%) cases had strong intensity of anti EP3 antibody staining in tumour tissue, *n* = 17 (37%) cases showed moderate intensity, and *n* = 15 (32.6%) cases showed weak intensity. *Conclusion*. Prostanoid EP3 receptors are widely but variably expressed in OSCC. Most of well differentiated OSCC cases show a moderate to strong expression of EP3 receptors. However, insignificant statistical relation to histological grades of OSCC has been observed. This might be due to small sample size of the study.

## 1. Introduction

Globally, oral cancer is the eighth most common cause of cancer-related deaths, but in spite of this fact most of the people are unaware of its presence [1]. Squamous cell carcinomas (SCC) comprise more than 90% of these oral cancers arising in the mucous membranes of the oral cavity and oropharynx [2]. OSCC is manifested in various clinical forms. It may resemble leukoplakia, verrucous leukoplakia, or an erythroleukoplakia. The erythroplakia may eventually develop into a necrotic ulcer with irregular, raised indurated edges or as an exophytic broad-based mass with warty surface. When traumatised, OSCC bleeds easily and frequently becomes secondarily infected at the surface. It is usually painless unless secondarily infected. Larger lesions can interfere with normal speech, chewing, or swallowing [3, 4].

OSCC arises from various molecular pathways that progress from the mutual effects of susceptibility and vulnerability to environmental carcinogens. These carcinogens are snuff, chemical carcinogens, alcohol, microorganisms, and ultraviolet or ionizing radiation. Damage to genes and chromosomes may occur due to prolonged exposure to these carcinogens [5].

For decades, no change in mortality rate has been observed. Even with advancements in surgery and radiotherapy, mortality still is very high with a 5-year survival rate of only 50% [6]. Currently, targeted molecular therapy such as monoclonal antibody therapy and gene therapy has been applied to patients with oral cancer. This treatment modality has limited or minimal side effects in normal cells of the body, in contrast to surgery, chemotherapy, and radiotherapy. The important molecular targets in cancer chemotherapy are progesterone receptor, cyclooxygenase-2 (COX-2), peroxisome proliferator-activated receptor (PPAR), and epidermal growth factor receptor (EGFR). These molecular targets are related with the proliferation and the differentiation of OSCC [7].

Among the prostanoids, the E-type PGs, particularly PGE2 derived from arachidonic acid, are produced in the body. PGE2 is found in most animal species and exhibits the multipurpose actions. There are four different receptors labelled EP1, EP2, EP3, and EP4 for the E-type PGs [8]. The probable involvement of PGs in the carcinogenesis is reinforced by the presence of enhanced expression of cyclooxygenase-2 in both premalignant and malignant tissues. There is also evidence that growth factors, cytokines, oncogenes, and tumour promoters stimulate COX-2 transcription via protein kinase C (PKC) and Ras-mediated signaling [9]. Further evidence of the role of PGs in carcinogenesis is evident from the plentiful epidemiological studies showing that continued consumption of drugs, especially aspirin, prevents the development of cancer [10, 11]. PGs play a vital role in numerous mechanisms that have been involved in carcinogenesis such as cell proliferation, angiogenesis, apoptosis, and mutagenesis [12]. Regardless of the fact that prostaglandin E2 is a notable carcinogenic agent and that PGE2 modifiers have a potential beneficial role, very few studies have so far fully investigated the components that are involved in synthesis and action of PGE2. The objective of the present study was to carry out a descriptive analysis of the expression of the EP3 receptors of PGE2 in different histological grades of OSCC. To our knowledge no such study elaborating the relationship between expression of PGE2 receptors EP3 in oral squamous cell carcinomas of various histological differentiation and adjacent normal tissue has so far been carried out in Pakistan. The results of this study may also help in assessing the presumptive prognostic role of PGE2 receptors EP3 in oral squamous cell carcinoma especially in terms of targeted therapy.

## 2. Methodology

### 2.1. Materials and Methods

After informed consent, biopsy specimens from 46 patients having undergone various diagnostic and/or surgical resection procedures for OSCC were acquired from Maxillofacial Surgical Department of NID, Multan. Sociodemographic information (name, age, gender, occupation, full address, history of smoking or using any form of smokeless tobacco, and family history of any cancer and oral cancer) was obtained along with relevant clinical, laboratory, and radiological information. To confirm the diagnosis 5 *μ*m thick, paraffin embedded sections were cut and mounted on glass slides and sections were stained with haematoxylin and eosin stain and examined by light microscope. Anneroth's histological grading system had been used to grade OSCC cases on H&E staining [13].

### 2.2. Immunohistochemistry

About 4 *μ*m thick sections were cut from all specimens and mounted on positively charged glass slides. Sections were deparaffinized in xylene and rehydrated in graded ethyl alcohol, followed by immersion in citrate buffer solution of pH 6.0, and were put in the microwave oven before staining procedures. For immunostaining, Universal Kit (Lab Vision) employing the streptavidin biotin system was used to carry out the peroxidase-antiperoxidase method of immunohistochemistry staining. Sections were then incubated with a primary polyclonal anti-EP3 receptor antibody (anti-PTGER3 antibody; ABCAM) and DAB chromogen was applied to the sections followed by counterstaining with haematoxylin [14].

The intensity and proportion of positive tumour cells of the EP3 immunohistochemical staining were evaluated using a light microscope. The intensity of cytoplasmic staining was graded from 0 to 3 (0: negative, 1: weak, 2: moderate, and 3: strong) and the tumour was also scored according to the proportion of positive tumour cells (0: 0–10% positive cells, 1: 11–25% positive cells, 2: 26–50% positive cells, 3: 51–75% positive cells, and 4: 76–100% positive cells) [15, 16]. The data was entered and analysed using SPSS 20.0. Data was expressed as mean ± SD. Comparisons between clinical and microscopic parameters were performed with the sample *t*-test. A difference of *P* < 0.05 was considered to be significant. The present study was certified by the Ethical Review Committee and Advanced Studies and Research Board of University of Health Sciences Lahore.

## 3. Results

A total of 46 cases of OSCC had been collected in accordance with inclusion and exclusion criteria. The overall mean age was 52.74 ± 14.14. The age range in males was 30 to 80 years (mean age 53.48 ± 13.27) while in females the age range was 25 to 95 years (mean age 51.68 ± 15.61). This data shows that age of incidence was similar in both genders; however, most of the patients were in the range of 40 to 70 years of age (*n* = 36). In the present study male to female ratio was found to be 1.4 : 1 (Table 1).

**Figure 1:**
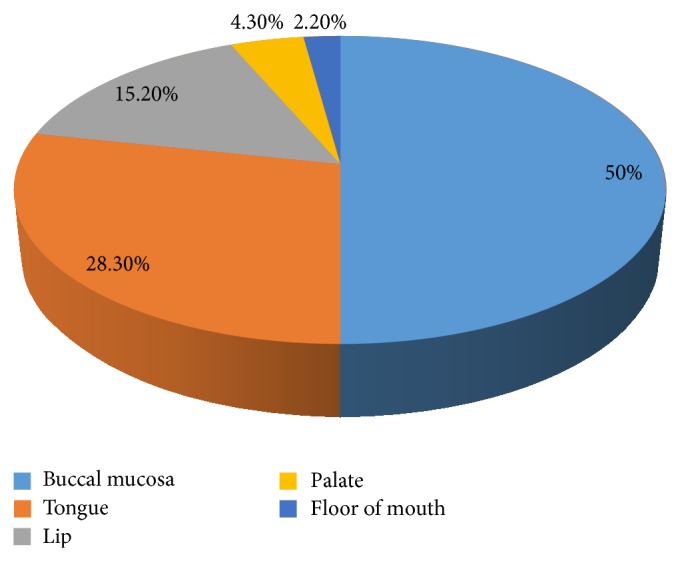
Distribution of OSCC cases at different sites of oral cavity.

Patients clinically presented most commonly with ulceration which was noted in 35 (76.1%) cases, fungating mass in 8 (17.4%) cases, and plaque-like lesion in 3 (6.5%) cases. Regarding the site of involvement of oral cavity, the occurrence of OSCC was found significantly higher on buccal mucosa where 23 (50%) cases were present. There were 13 (28.3%) cases that involved the tongue (mostly lateral border of tongue). When site of specimen was corelated with gender, the most number of cases in both genders were located on buccal mucosa but in females *n* = 9 (47%) cases were located on tongue while in males only *n* = 4 (14.8%) cases were located on tongue. When *t*-test was applied to observe the statistical relation it was significant (*P* = 0.01) (Figure 1).

A total of 60.9% were well differentiated (WD) (Figures 9 and 11), 32.6% were moderately differentiated (MD) (Figure 13), and only 6.5% were poorly differentiated (PD) cases of OSCC (Figure 15). Lymphovascular invasion was present in 61.8% of cases.

**Figure 2:**
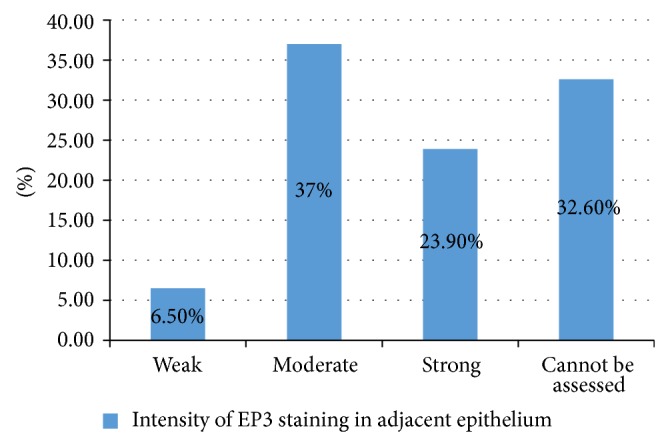
Intensity of EP3 staining in adjacent epithelium.

Among 46 cases of OSCC, 31 cases had attached nontumorous epithelium. Out of these 31 cases of OSCC with attached epithelium, 11 (23.9%) had strong intensity (Score +3) of EP3 antibody staining, 17 (37%) had moderate intensity (Score +2) of antibody staining, and only 3 (6.5%) cases had weak intensity (Score +1) of EP3 antibody staining (Figures 2, 5, 6, 7 and 8).

**Figure 3:**
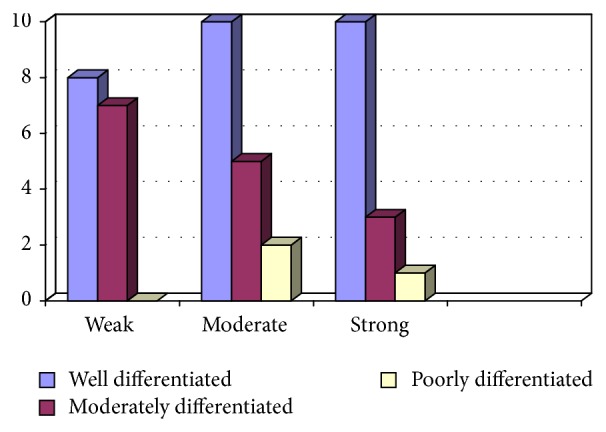
Comparison between intensity of EP3 staining in tumour tissue and histological grades of OSCC.

Among 46 cases of OSCC, 14 (30.4%) cases had strong intensity (+3) of anti-EP3 antibody staining in tumour tissue, 17 (37%) cases showed moderate intensity (+2), and 15 (32.6%) cases showed weak intensity (+1) (Figure 3).

The statistical relation between intensity of antibody staining and histological grade of OSCC was insignificant (*P* = 0.486).  Table 2 shows the comparison between intensity of antibody staining and histological grade of OSCC (Table 2).

The number of cases showing weak intensity EP3 antibody in adjacent epithelium was *n* = 3 (6.5%) while the number of cases showing weak intensity in tumorous tissue was *n* = 15 (32.6%) in the present study (Figures 14 and 16). This difference in intensity of EP3 antibody staining in both of these tissues is statistically significant (*P* = 0.042).

As already stated above that a total of 31 cases had attached nontumorous epithelium, 11 (23.9%) cases had 76–100% (Score +4) positive epithelial cells for EP3 antibody, 11 (23.9%) cases had 51–75% (Score +3) positive epithelial cells, 26–50% (Score +2) epithelial cells were positive in 8 (17.4%) cases, and only 1 (2.2%) had 11–25% (Score +1) positive epithelial cells.

**Figure 4:**
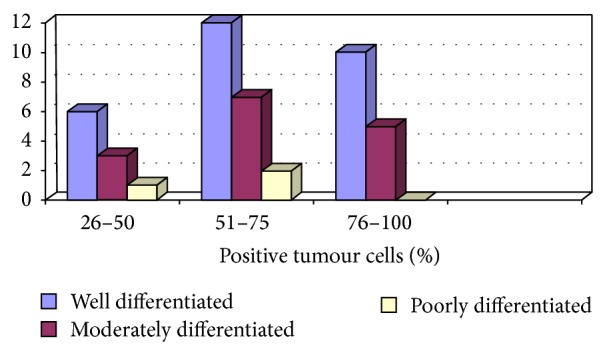
Comparison between histological grade and proportion of positive tumour cells.

**Figure 5:**
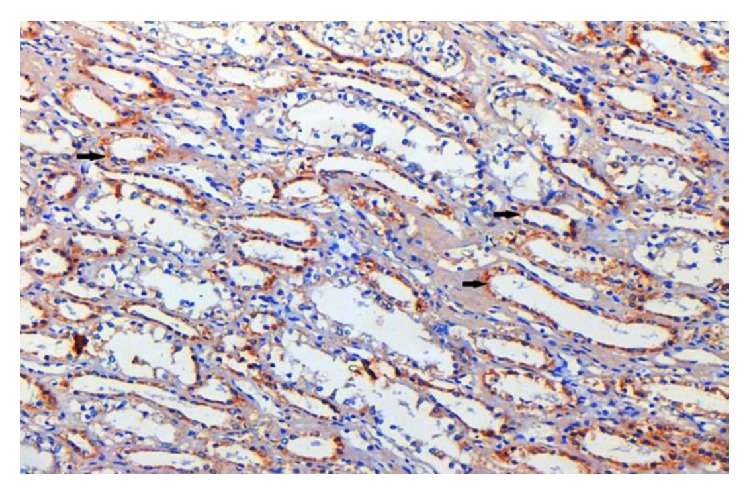
Human kidney tubules (control tissue) lining epithelial cells IHC staining (EP3 IHC, 20x).

**Figure 6:**
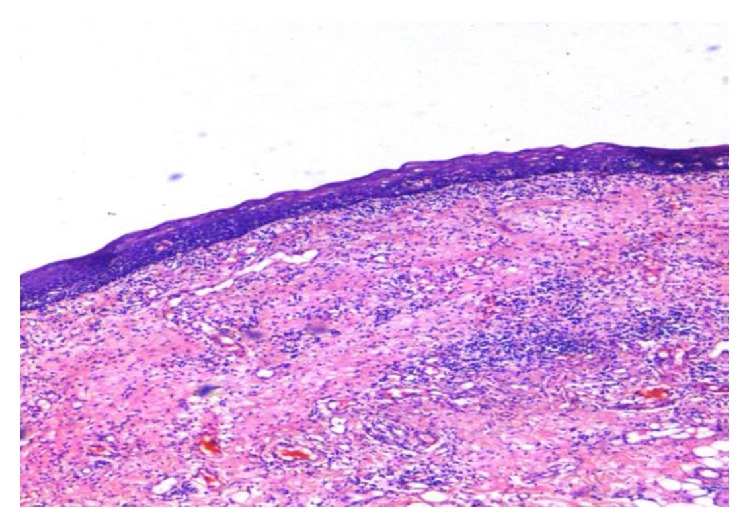
Oral epithelium with atrophy of rete ridges and inflammatory infiltrate in underlying connective tissue (H&E, 10x).

**Figure 7:**
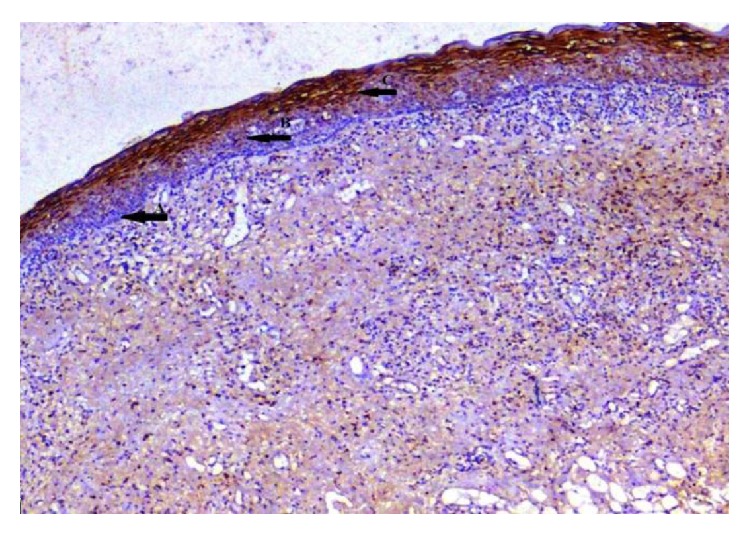
Oral epithelium showing differing staining intensity: (↑A) basal epithelial cells negative staining, (↑B) moderate, and (↑C) strong (EP3 IHC, 10x).

**Figure 8:**
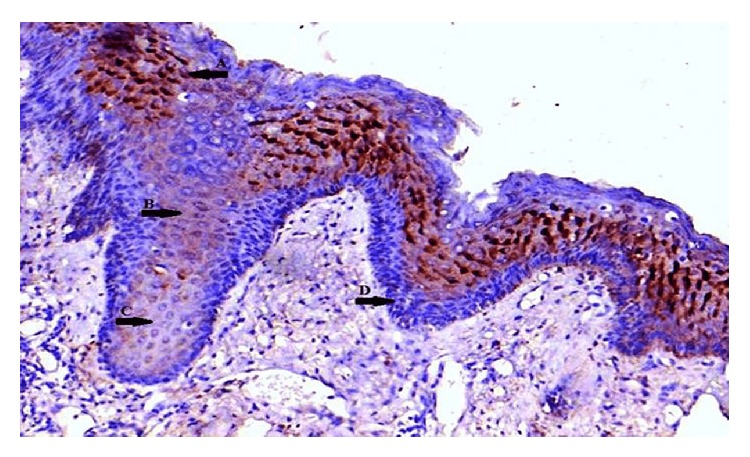
Normal oral epithelium showing differing staining intensity: (↑A) strong, (↑B) moderate, (↑C) weak, and (↑D) basal epithelial cells negative staining (EP3 IHC, 20x).

**Figure 9:**
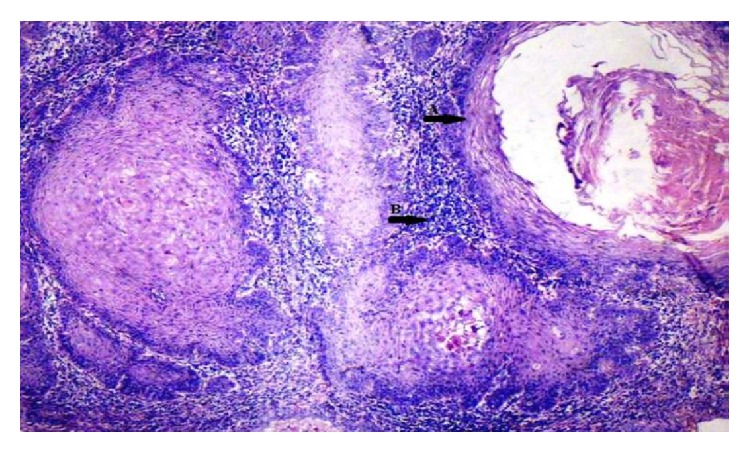
Photomicrograph of well differentiated OSCC: (↑A) keratin pearl, (↑B) intense inflammatory infiltrate (H&E, 20x).

Among 46 cases of OSCC, 15 (32.6%) cases had 76–100% (Score +4) positive tumour cells for EP3 antibody, 21 (45.7%) cases had 51–75% (Score +3) positive tumour cells, and 10 (21.7%) cases had 26–50% (Score +2) positive tumour cells (Figure 4).

When we applied *t*-test to observe the statistical relation between histological grade and proportion of positive tumour cells for EP3 antibody, it was found to be insignificant (*P* = 0.843). It was also insignificant when it was applied to find the relation between proportion of positive adjacent epithelial and tumour cells for EP3 antibody (*P* = 0.213).

**Figure 10:**
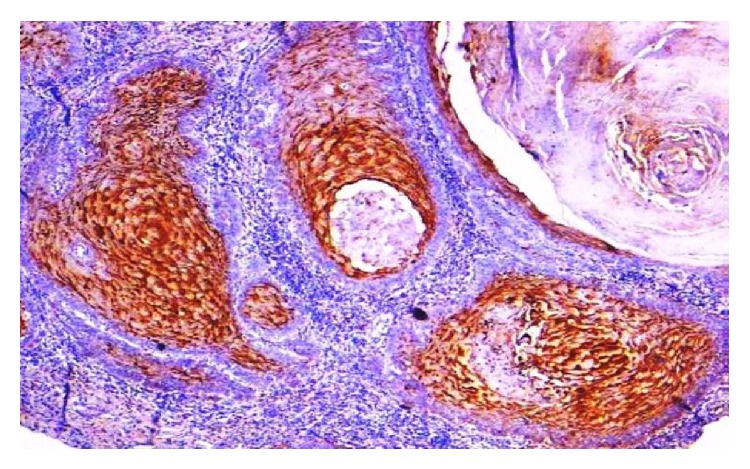
Photomicrograph of well differentiated OSCC showing strong intensity of staining (EP3 IHC, 20x).

**Figure 11:**
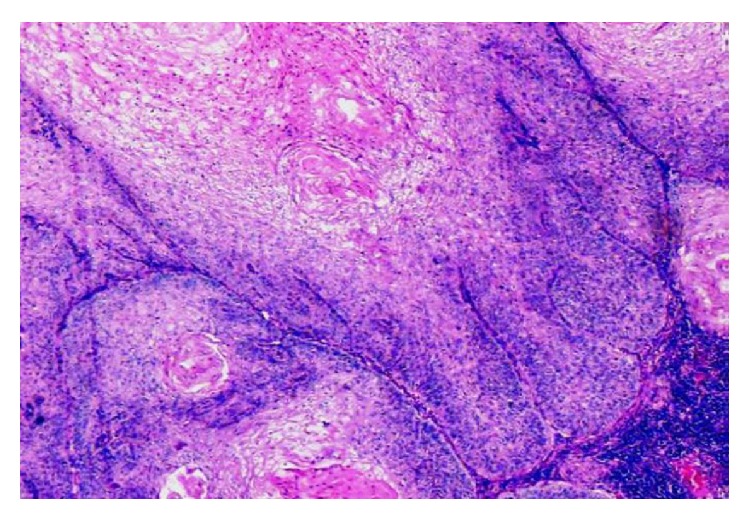
Verrucous variant of SCC with tonguelike projections, abundant keratinization, and mild degree of lymphocytic infiltrate (H&E, 20x).

**Figure 12:**
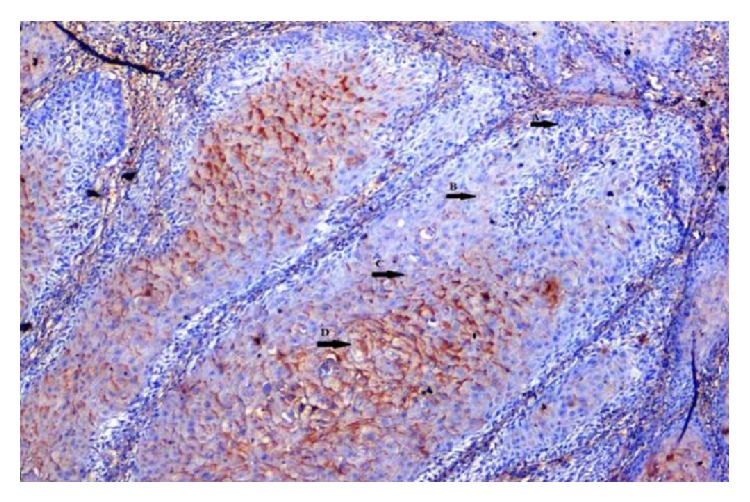
Photomicrograph of verrucous variant of OSCC showing differing staining intensity: (↑A) negative, (↑B) weak, (↑C) moderate, and (↑D) strong (EP3 IHC, 20x).

**Figure 13:**
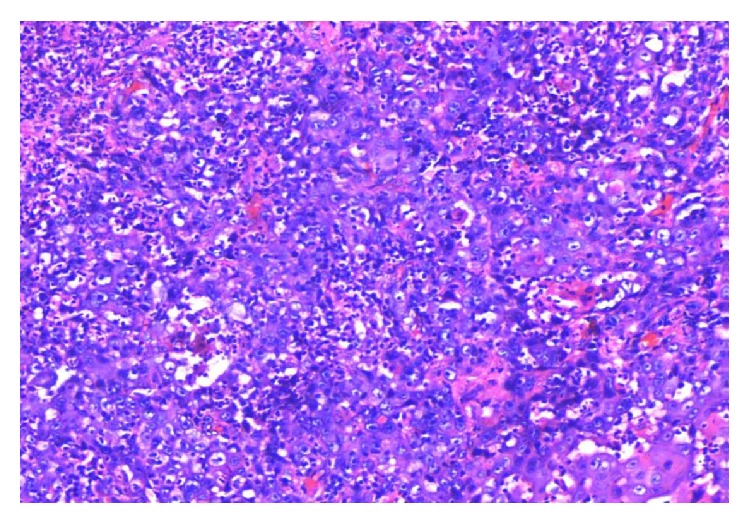
Photomicrograph of moderately differentiated OSCC (H&E, 20x).

**Figure 14:**
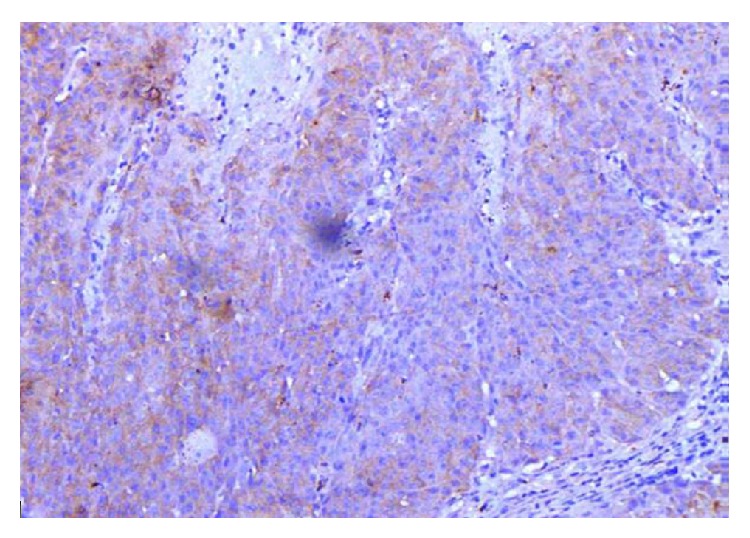
Photomicrograph of moderately differentiated OSCC showing weak to moderate intensity of staining (EP3 IHC, 20x).

**Figure 15:**
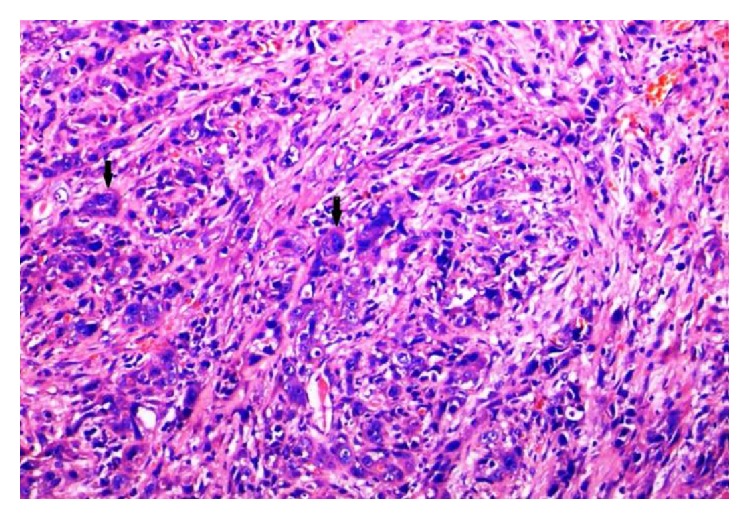
Photomicrograph of poorly differentiated OSCC with high degree of cytological and nuclear pleomorphism (↑) (H&E, 20x).

**Figure 16:**
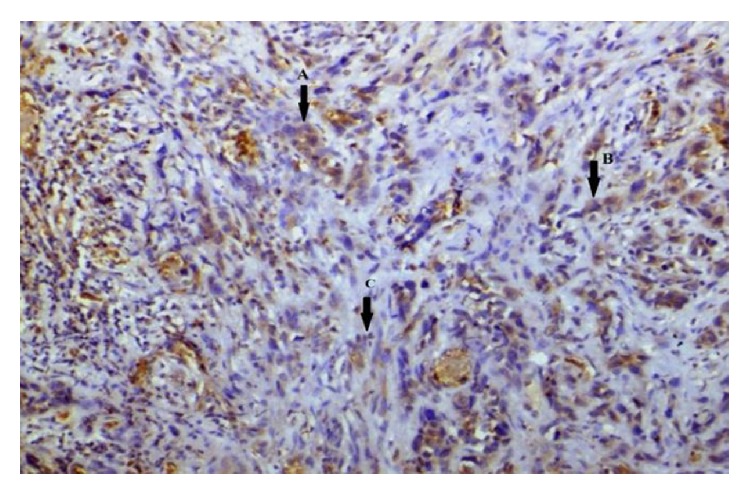
Photomicrograph of poorly differentiated OSCC showing weak intensity of staining (EP3 IHC, 20x).

In summary, all 46 cases of OSCC were positive for EP3 receptor antibody. The maximum intensity and proportion score of EP3 in WDOSCC were +3 (35.5%) and +3 (45.6%); similarly 46.6% of MD exhibited +1 and +3, while 66% of each of PD tumours demonstrates +2 and +3 of intensity and proportion score, respectively. So most cases of well differentiated OSCC (71%) showed moderate to strong intensity of staining (*P* = 0.486). A total of 31 cases had attached nontumorous epithelium, 54.8% showing (+2) intensity score, 35.4% cases showing (+4) proportion score for EP3 antibody (Figures 10, 12, 14 and 16).

## 4. Discussion

The likelihood of developing oral SCC increases with the period of exposure to risk factors and increasing age adds to the dimension of the mutagenic and epigenetic changes associated with age. In the present study 1.4 : 1 male to female ratio was noted, showing male predominance. The overall mean age was 52.74 ± 14.14. These findings are consistent with certain European studies which reported male predominance in patients of OSCC [17]. Most of local studies from Pakistan also reported male predominance [18, 19]. A morphological study of OSCC carried out in Pakistan by Ayaz et al. also reported 1.5 : 1 male to female ratio with 53 (SD ± 15.16) years of mean age of incidence [20]. The exception to gender related findings of this present study is a study from Lahore which reported female predominance with a ratio of 1.5 : 1 [21]. Another study carried out in India on 80 cases of OSCC reported a prevalence of cancer in 61.25% of males [22] and Yazdi and Khalili in their study in Iran on 48 cases of OSCC of tongue also reported male prevalence of 60.4%. The present study is also consistent with both of these studies. The high proportion of cases among men may be due to the high prevalence of snuff consumption habits in them, combined with cigarette smoking, whereas in our society females less commonly indulge in tobacco smoking [23].

Considering the site of involvement in the oral cavity, the occurrence of OSCC was significantly higher on buccal mucosa where 23 (50%) cases were present followed by 13 (28.3%) cases that involved the tongue (mostly lateral border of tongue). A study by Tahir et al. on OSCC reported results which were similar to the present study regarding the site of involvement. The most frequent location of OSCC in that study was also buccal mucosa (32.4%) (slightly higher in the present study which was 50%) followed by tongue (21.6%); this value is also comparable to the present study (28.3%) [24]. Many studies carried out in the USA and Europe reported quite different findings. In Western countries OSCC involves the tongue in 20%–40% of cases and the floor of the mouth in 15%–20% of the cases, and together these sites represent approximately 50% of all cases of OSCC. The gums, palate, retromolar area, and the buccal and labial mucosa are oral sites affected less frequently [4, 25, 26].

Tumours can grow to a size that exceeds its blood supply, leading to tumour necrosis and ulceration. In the present study the most frequent presenting symptom was ulcerating lesion on buccal mucosa (*P* = 0.049). This is comparable to another study carried out in Pakistan in the recent past in which the most common clinical presentation of OSCC was a nonhealing indurated ulcer (51.4%) and it was also significantly common on the buccal mucosa (*P* = 0.001) [24]. Another study carried out in Canada by Mirbod and his coworker also reported that ulceration was the most common finding in OSCC patients; this finding is consistent with the present study [27].

In the current study out of 46 cases, lymphovascular invasion was found in 33 (71.7%) while 13 cases showed no lymphovascular invasion. In another study carried out in Japan Nomura et al. reported 57.5% lymphovascular invasion in cases of OSCC, which is slightly lower than the present study [28]. In the present study, among all the 46 cases of OSCC, 31 had attached nontumorous epithelium. Adjacent epithelium showed reactivity of varying intensity and proportion in all of these 31 cases of OSCC with attached epithelium in the present study. A few studies carried out in Japan reported that EP3 receptor is extensively expressed in all the human body organ systems. More or less all tissues in mice and rats express its mRNA [8]. Shoji and his associates also reported that EP3 receptors may play a vital role in skin carcinogenesis. They demonstrated this fact in EP3 receptors knockout mice by observing two-stage skin carcinogenesis [29]. All these studies validate our findings that squamous epithelium expresses the EP3 receptors normally. Similarly, in present study, all 46 cases of OSCC were variably positive for EP3 receptor antibody. As shown in Results, the number of cases showing weak intensity of EP3 antibody staining in attached epithelium was 3 (6.5%) while the number of cases showing weak intensity in tumorous tissue was 15 (32.6%) in the present study which is five times higher frequency of intensity of EP3 staining in tumorous tissue than frequency in attached epithelium. This difference in intensity of EP3 antibody staining in both of these tissues is statistically significant (*P* = 0.042). This finding suggests that as the squamous epithelial cell becomes less differentiated it loses its ability to express prostanoid EP3 receptors. This was also observed during the histological examination of OSCC cases stained with EP3 antibody: as the squamous cell is more differentiated it shows more intense staining and as the cell gradually loses its differentiation, it also loses its property to express prostanoid EP3 receptors and the intensity of staining with EP3 antibody becomes weak. When we applied Fisher's exact test to observe the statistical relation between intensity of antibody staining and histological grade of OSCC, it was found to be insignificant (*P* = 0.486), because overall intensity of staining was randomly distributed among different grades of OSCC.

A similar study carried out in Brazil by Ryott et al. reported that 94% of cases of OSCC were positive for EP3 receptor antibody and most of the cases in that study were of moderately differentiated histological grade [15]. While in lung and colon carcinogenesis the role of prostaglandin E2 receptors is well established by many researches, a small number of studies have assessed their involvement in HNSCC and described that EP3 and EP4 receptors were expressed in cancer cell lines but Very weak EP1 and EP2 receptors expression was noticed [30]. Hoshikawa et al. also showed antiproliferative effects of EP3 receptor signalling pathway in cancer lines and EP3 receptor antagonist decreases the PGE2 level in vitro and stops the cell cycle in Go/G1 phase of cell cycle [31]. The growth and proliferation of head and neck squamous cell carcinoma cells were shown to reduce by the application of EP3 receptor antagonists [31].

Knowledge of the molecular mechanisms underlying dysregulated growth and progression of OSCC may provide a rational basis for exploring new treatment modalities and can prove valuable in the development of new molecular therapies and targeting tools. Since PGE2 is likely to pile up at high levels in the tumour microenvironment and can be produced by the action of the COX-2 expressed in both cancer and stromal cells [15], PGE2 may play an important role in OSCC carcinogenesis via acting on EP3 receptors. Prostaglandin E2 can also play a vital role in increasing the proliferative capability of OSCC cell lines. As depicted from these studies, targeted therapies against prostaglandin E2 and EP receptors may represent valuable adjuvant modalities in treatment of the initial phases of development of OSCC.

To our knowledge no such documented immunohistochemical study elaborating the role of prostanoid receptor EP3 in OSCC has been carried in Pakistan or any other South Asian country so there is a lack of data comparison in this aspect. Further studies on large sample size are now needed to elucidate the underlying molecular and genetic mechanisms to assess the tumour related effects of PGE2 and its receptors in vivo. This area of future research undoubtedly will further improve our understanding of carcinogenesis of OSCC and the role played by EP3 receptors and prostaglandin E2.

## 5. Conclusion

Prostanoid EP3 receptors are widely but variably expressed in OSCC. Most of well differentiated OSCC cases show a moderate to strong expression of EP3 receptors (Figure 10). However insignificant statistical relation to histological grades of OSCC has been observed. This might be due to small sample size of the study. By acting on EP3 receptors expressed on OSCC cells, PGE2 may contribute to increase of OSCC cell proliferative potential. In this regard, PGE2 blocking therapies, as well as EP3-specific treatment modalities, may represent helpful approaches as adjuvants in OSCC treatment for patients at the initial stages of OSCC development. Immunohistochemical findings of the present study may predict that expression of EP3 receptors in OSCC may play a significant role in the carcinogenic progression of squamous epithelial cells. Further studies are needed on a larger sample size that may focus on molecular and genetic pathways to completely define the role of PGE2 receptors in oral carcinogenesis.

## Figures and Tables

**Table 1:** Gender frequency in study cases.

Gender	Frequency	Percent
Male	27	58.7%
Female	19	41.3%

Total	46	100.0%

**Table 2:** Proportions of positive adjacent epithelial cells.

Proportion of positive epithelial cells	Frequency	Percent
11–25% positive epithelial cells (Score +1)	1	2.2%
26–50% positive epithelial cells (Score +2)	8	17.4%
51–75% positive epithelial cells (Score +3)	11	23.9%
76–100% positive epithelial cells (Score +4)	11	23.9%
Cannot be assessed	15	32.6%

Total	46	
